# Experimental Investigation on the Effect of Dry Ice Compression on the Poisson Ratio

**DOI:** 10.3390/ma15041555

**Published:** 2022-02-18

**Authors:** Aleksandra Biszczanik, Jan Górecki, Mateusz Kukla, Krzysztof Wałęsa, Dominik Wojtkowiak

**Affiliations:** Institute of Machine Design, Poznan University of Technology, Piotrowo 3, 61-138 Poznań, Poland; mateusz.kukla@put.poznan.pl (M.K.); krzysztof.walesa@put.poznan.pl (K.W.); dominik.wojtkowiak@put.poznan.pl (D.W.)

**Keywords:** Poisson ratio, compression, densification, extrusion, dry ice, carbon dioxide (CO_2_)

## Abstract

In the processing of waste materials, attention must be given to the efficient use of energy. The pelletization of dry ice is a good example of such processes. A literature review shows that in the pelletizers available on the market, the force applied in the process is excessive. As a result, the efficiency of the utilization of inputs, including electricity and carbon dioxide, is at a very low level. This article presents the results of experimental research on the effect of the degree of dry ice compression on the value of the Poisson ratio. The first part of this article presents the research methodology and a description of the test stand, developed specifically for this research, bearing in mind the unique properties of carbon dioxide in the solid state. The results presented show the behavior of dry ice during compression in a rectangular chamber for different final densities of the finished product. As a result, it is possible to determine the values of the Poisson ratio as a function of density, using for this purpose four mathematical models. The findings of this research may be useful for research work focused on the further development of this process, such as by using the Drucker–Prager/Cap numerical model to optimize the geometric parameters of the parts and components of the main unit of the machine used in the extrusion process of dry ice.

## 1. Introduction

The available research reflects a growing interest in the use of numerical testing techniques in the planning process of various production processes, such as powder densification [1,2,3], the compaction of saw dust [4] or dry ice blasting [5]. This stems from the possibility of reducing the implementation costs related to the preparation of the prototype and carrying out the tests needed in the period before starting production. As a result, it is possible to improve the efficiency of production processes at the planning stage and in the process of designing relevant machines. The improved efficiency of processes in the area of the utilization of raw materials contributes to the reduction of production waste. This should be understood as a use of raw materials that can be considered both effective and efficient, which makes it possible to minimize losses along the process of transporting and using the materials in production—for example, natural gas liquids [6]. There is also material efficiency research aimed at improving design and recycling processes, such as [7]. This can be especially illustrated by the example of rare earth metals, the recycling rates of which are estimated at less than one percent [8]. A good example of production waste reduction is the use of polymer materials in additive manufacturing processes, such as polylactic acid (PLA), acrylonitrile butadiene styrene (ABS), poly (vinyl alcohol) (PVA), and a number of others. The use of 3D printing technology generates less production waste than the subtractive methods of production. As a result, it is characterized by a number of advantages, including improved resource efficiency concerning both production and manufacturing processes [9].

However, it should be noted that despite efforts to manage waste generated throughout the product life cycle, the current level of techniques does not allow zero waste production, as illustrated by the example of a wind turbine in [10]. Despite generating relatively clean and emission-free electricity, building a wind power plant consumes a significant amount of energy and materials, which in turn generate waste during the manufacturing process. Furthermore, there is also the issue of the withdrawal from use of recycling materials from wind farms. In addition to the easily reusable steel from which the base of the wind turbine is made, there are the blades of wind turbines, which are made of lightweight, composite polymer materials. They are characterized by a large volume and a number of difficulties during recycling [11]. Therefore, it is key to continue research with the objective of improving the performance of machines and the efficiency of recovery and recycling of waste materials [11].

Carbon dioxide is an example of such waste material generated in production processes. It is a greenhouse gas that, according to the reports of the Intergovernmental Panel on Climate Change, is directly responsible for climate change on Earth [12]. While the burning of fossil fuels leads to high levels of CO_2_ in the Earth’s atmosphere, amounting to approximately 60%, the main contributors to the increase in carbon dioxide emissions into the atmosphere are agrochemical, as well as pharmaceutical plants [13]. Ulhman et al. pointed out that ammonia production is the source of carbon dioxide emissions with the highest share in its global emissions [14]. Ammonia plants are very often equipped with carbon dioxide capture systems, making it possible to use this gas as a raw material in other production processes. Crude oil and gas refineries come next in terms of the percentage of total carbon dioxide emissions [15].

In ammonia factories, carbon dioxide is not a key production material; the volume of the recovered material exceeds the internal needs and the surplus amount cannot be reused on site. Therefore, this surplus material is supplied to whoever needs it. To facilitate transport and storage, carbon dioxide is compressed to 2 MPa, resulting in its condensation [14]. Supplied in this form, the material is then reused, for instance, for the production of solid carbon dioxide, obtained by expansion to atmospheric pressure. With a solidification temperature of −78.5 °C [16], this material sublimes in ambient conditions. These peculiar properties of the material led to its common name, ‘dry ice’. However, carbon dioxide can also be transported through pipelines in its supercritical phase or in the sub-cooled phase. Both of those states are high-density fluids [17].

The rate of dry ice sublimation is a challenge for its efficient utilization. Therefore, a treatment is applied to reduce the sublimation rate, which is reduction of the surface on which the phase transition occurs. This can be achieved, for example, by compressing it into pellets.

In this form, CO_2_ has found a number of applications, including in the transport of materials and products that require reduced temperature values [18]. Due to its non-flammability and non-toxicity (in this context), it is used as an environmentally friendly material [19], which allows it to be used, in certain cases, as an extinguishing agent, or in potentially explosive areas. As a material that is non-conductive and sublimating in natural conditions, it is also used as a surface-cleaning agent, especially in semiconductor devices and in the food industry [20], where its disinfecting properties are also applied [18]. It is an alternative to surface cleaning with the use of solvents [21]. Dry-ice blasting is usually carried out through the technique of blowing dry ice pellets under pressure. This process allows the waste-free cleaning of surfaces [22]. However, it involves significant energy losses resulting from the use of additional compressed air, and considerable noise; therefore the use of this cleaning technology may be limited [20]. In addition, carbon dioxide in high concentrations is dangerous to health, which makes it necessary to use it in well-ventilated rooms.

**Figure 1:**
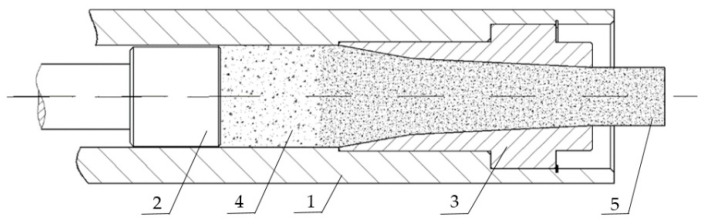
The main part of the piston-type pelletizer. 1—compaction chamber, 2—piston, 3—die, 4—dry ice before compression, 5—compressed dry ice.

Dry-ice pellets are made using pelletizers. Their task is to compact and extrude dry-ice snow. In this process, an appropriately densified material in the shape of pellets is obtained. The compression of the material reduces the surface area of the dry ice, slowing the loss of mass due to sublimation. Compaction processes and techniques have been extensively described in previous studies. For loose and commuted materials, piston, worm or roller extrusion mechanism methods are used, depending on their properties and energy consumption. It is worth mentioning that roller presses have relatively lower energy consumption compared to the other two methods (worm and piston mechanisms) [23]. Examples of the use of pelletizers in worm and roller technology include, among others, the compaction of wood sawdust, bronze and other metals, and swarf [23]. Roller machines cause a noticeable and uneven increase in the temperature value on the briquette surface [24], which is not desirable in the case of dry ice, for which an increase in the ambient temperature means an increase in the intensity of the sublimation process. In the case of dry ice, the only option is to produce pellets using reciprocating machines. Such machines are equipped with a reciprocating system, in which the dry ice snow is compacted and then pushed through a single- or multi-channel die. The shape of the extrusion die significantly affects the extrusion pressures used in this process [25], thus increasing power demand. An example of the extrusion system is shown in Figure 1.

Górecki et al. demonstrated that the extrusion processes are characterized by low efficiency and levels of work load that are too high [26]. This results in an excessive consumption of electricity and raw materials [27]. The geometrical parameters of the elements of the main unit are relevant in this respect. Therefore, the authors undertook research to develop solutions to improve the efficiency of such machines, both existing and new. In the available research, works can be found that are related to the use of the finite element method to analyse the working load values in compaction processes using the Cam Clay [28], Drucker Prager/Cap [29], and Mohr–Coulomb models [30].

During the simulated compaction process, the material changes its structure from comminuted, through porous, to crystalline. Therefore, to carry out the analyses with the use of the models mentioned above, it was necessary to define the characteristics of the change in the mechanical properties of the material in question, as a function of its degree of compaction. Diarra et al. and Han et al. indicate that knowledge of the characteristics of the change in the value of the Poisson’s ratio is necessary during numerical tests related to, for example, phenomenal-elastic elastically plastic models such as the Drucker–Prager/Cap or the Modified Cam-Clay model. Numerical simulations—with the use of the indicated models—allow the commencement of studies related to the improvement of the efficiency of the compaction process. As indicated in a previous study, limiting the compaction stress to the necessary minimum not only reduces electricity consumption but also improves the efficiency of material use [31].

Due to the peculiar properties of dry ice, it was necessary to develop an appropriate test method and a test stand suitable for testing at a reduced temperature.

The article presents the test method and results of the research used for determining the characteristics of the change in the Poisson ratio as a function of the degree of dry ice densification. To determine the mathematical function describing the characteristics of the change in the Poisson ratio *ν* as a function of the density *ρ*, four mathematical models were proposed, which were compared with each other in terms of correlation with the experimental results and the standard error.

## 2. Materials and Methods

The purpose of this study was to determine the Poisson ratio of dry ice as a function of its density. The input material for the testing was pulverized dry ice with a bulk density of approximately 500 kg/m^3^, compressible to approximately 1625 kg/m^3^ [32]. The material was stored in an insulated container to reduce the sublimation due to the transfer of heat from the environment and to reduce the condensation and crystallization of the water vapour from the air.

The value of the Poisson ratio *ν* for a different degree of compression of the isotropic material was determined using the methodology described in [33]. It assumes that when the material is compacted in a closed chamber, the transverse strains *ε_x_* and *ε_y_* are equal to 0, and the strains *σ_x_* and *σ_y_* acting on the chamber’s side walls are equal to each other. With such assumptions, Equation (1) can be transformed into Equation (2). Thus, with the known *σ_x_* and *σ_z_* values, it is now possible to determine the value of *ν*.

(1) $$\varepsilon_{y}=\frac{1}{E}\left(\sigma_{y}-\nu \left(\sigma_{z}+\sigma_{x}\right)\right)=0$$

(2) $$\nu =\frac{\sigma_{x}}{\sigma_{z}+\sigma_{x}}$$

**Figure 2:**
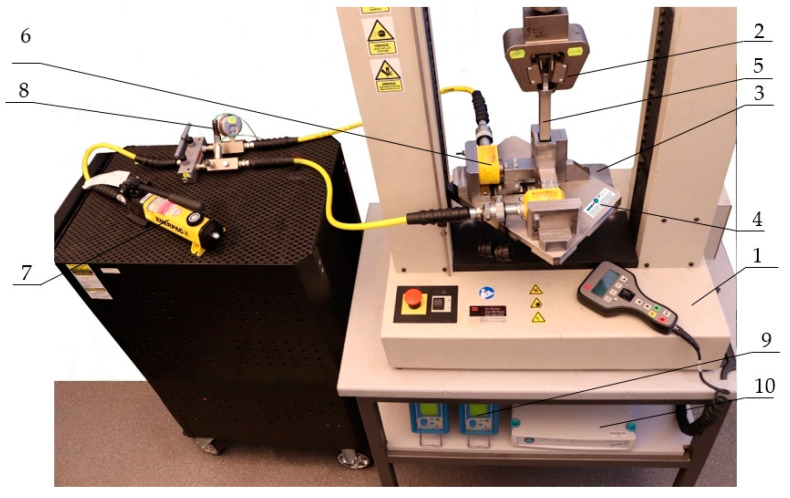
The measuring system for triaxial compression of dry ice specimens: 1—MTS Insight 50 kN universal testing machine; 2—grip; 3—table; 4—stand for triaxial compression; 5—stand piston; 6—ENERPAC hydraulic cylinder; 7—ENERPAC hydraulic pump; 8—pressure sensor; 9—Kistler charge amplifier; 10—Spider 8 measuring amplifier.

To determine the stress values for different levels of compaction, the authors used the measuring system shown in Figure 2, composed of the MTS Insight 50 kN (MTS Systems Corporation, Eden Prairie, MN, USA (MTS Systems GmbH, Berlin, Germany)) universal testing machine, a computer with software Test Works 4 (MTS Systems Corporation, Eden Prairie, MN, USA (MTS Systems GmbH, Berlin, Germany)) to control the machine, a specially designed stand for triaxial compression of specimens, a portable hydraulic system consisting of two ENERPAC RSM100 cylinders (ENERPAC, Enerpac Eastern Europe Sp. z o.o., Zabierzów, Poland) and an ENERPAC P141 hydraulic hand pump (ENERPAC, Enerpac Eastern Europe Sp. z o.o., Zabierzów, Poland) with a maximum pressure of 70 MPa, two Kistler 5015A1000 Charge Meter (Kistler Group, Kistler Eastern Europe s.r.o., Praha, Czech Republic) laboratory charge amplifiers fitted with sensors to measure the pressure in the system, a Spider 8 measuring amplifier (Hottinger Baldwin Messtechnik GmbH (HBM), Darmstadt, Germany), and a computer with HBM Catman Easy version 3.5 software (Hottinger Baldwin Messtechnik GmbH (HBM), Darmstadt, Germany) for signal recording. 

The tests were carried out using the MTS Insight universal tester with 50 kN force capacity equipped with a strain gauge sensor for force measurement and a displacement transducer, both 0.5 accuracy classes. A testing stand was installed between the grip and the machine table for triaxial compression of the specimen (Figure 2, label 4) to determine the stresses acting on the individual walls of the sample, which were needed to determine the Poisson ratio. The set of devices shown in Figure 2 was used to carry out the tests, during which it was possible to record the force value applied to the piston (Figure 2, label 5) as a function of its displacement, as well as to measure the pressure values *p_x_* and *p*_y_ in the hydraulic systems of both cylinders. The output signals from the sensors of the MTS machine were transmitted to the HBM Spider 8 measuring amplifier, while signal from the pressure sensor was sent first to the Kistler charge amplifier, and then to the Spider 8 measuring amplifier, from which the data were read by HBM’s Catman Easy program. The output signal values were acquired and processed with a frequency of 100 Hz. The PCB10000-1 balance (manufactured by KERN & Sohn GmbH, Balingen, Germany), with a measurement accuracy of up to 0.1 g, was used to measure the mass of the input material and the final mass of the specimen. The tests were performed in a room with an ambient air temperature of 18 °C and about 50% relative humidity.

**Figure 3:**
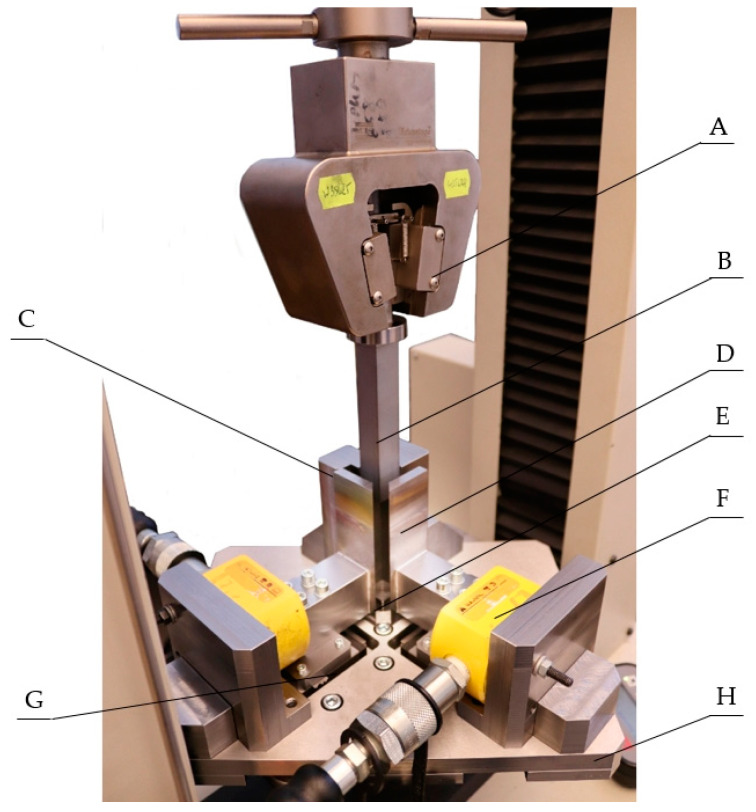
A, grip; B, piston; C, fixed chamber wall; D, sliding chamber wall; E, chamber interior; F, hydraulic cylinder; G, guideway; H, base plate of the stand.

In the first step, the triaxial compression stand (Figure 2, label 4) was stored in a container filled with dry ice to reduce its temperature. The purpose was to lower the sublimation rate of the material during the test. Before starting the series of tests, the stand (Figure 2, label 4) was cooled for 30 min. Subsequently, in the course of testing, the stand was cooled after two consecutive tests for a period of 15 min. Performing two consecutive trials took no more than 10 min, and the reassembly of the stand after taking it out of dry ice took approximately 2 min, the testing time was ca. 5 min, and the disassembly of the stand ca. 2 min. After cooling, the stand was reinstalled on the testing machine. The next step involved the mounting of the hydraulic cylinders (Figure 3, label F). The piston (Figure 3, label B) was mounted on the grip of the machine (Figure 3, label A), and then aligned relative to the side walls of the compaction chamber side walls (Figure 3, labels C and D). Pressure was applied to the cylinder via the hydraulic pump (Figure 2, label 2) and the cylinders by exerting pressure on the sliding side walls of the compaction chamber (Figure 3, label D), causing it to close and secure in position. The pressure values in the hydraulic cylinders (Figure 3, label F) were determined from the expected value of the initial final densities. Measurements were made for five hydraulic system pressure values, i.e.,: 0.3, 0.7, 1.3, 1.6 MPa, and 2.5 MPa. About 27 g of dry ice was fed into the chamber to obtain specimens ranging in height from 1.5 to 2 times the transverse dimension of the specimen, i.e., 20 mm. This made it possible to obtain specimens with uniform density distribution and isotropic mechanical properties [4]. Next, the program prepared with the Test Works 4 software was launched and the piston mounted in the MTS machine handle was moved downward at a speed of 5 mm/s, thus causing the dry ice to compact in the chamber until the measuring system recorded an increase in the pressure measured in the hydraulic system, which was caused by the pressure exerted by the specimen on the chamber walls. The pressure *p_1_* and *p_2_* in the individual hydraulic cylinder systems, the piston displacement, and the force exerted by the piston *F_Z_* were recorded at the same time. After stopping the machine, the pressure in the hydraulic system was reduced, the piston was pulled out of the chamber, the specimen was taken out, and its mass *m* was measured. Knowing the geometrical dimensions of the specimen after compaction, it was possible to determine the density of the pellets.

To determine the stress values *σ_x_* and *σ_y_*, it was necessary to determine the characteristics of both hydraulic cylinders. This was due to the increase in the viscosity of hydraulic oil as a result of the contact of the cylinder body with the cooled triaxial compression stand. The change in oil viscosity was associated with a change in the force on the cylinder piston. These characteristics served to obtain the following equation relating the force to pressure.

(3) $$F_{x}=1868.55p_{x}-777.39$$

where:

*p_x_*—pressure in the hydraulic system in Mpa;

*F_x_*—force exerted on the piston in N.

The determined value *F_x_* was converted into the stress value *σ_x_*. This was possible with the assumption that *ε_x_* = *ε_y_* = 0. Thus, the area of the side wall of the compaction chamber depended only on the height of the sample *h* and the transverse dimensions *a* and *b* were 20 mm. The height of the specimen was determined based on the output signal from the MTS Insigh 50 kN machine displacement sensor.

The above assumptions are represented by the following equation.

(4) $$\sigma_{x}=\frac{F_{x}}{A_{x}}=\frac{1868.55p_{x}-777.39}{a\cdot h}\;,$$

The value of the compressive stresses induced by the pressure of the piston on the dry ice was determined using the following equation:

(5) $$\sigma_{z}=\frac{F_{Z}}{A_{Z}}=\frac{F_{z}}{a\cdot b}\;,$$

where:

*F_Z_*—force exerted by the strain gauge sensor of the MTS Insight 50 kN system;

*A_Z_*—surface area of the specimen in the XY plane.

To reduce the error due to the frictional resistance on the side walls of the compaction chamber, additional tests were carried out to determine the value of the movement of the resistance force during the piston movement inside the empty compaction chamber. In the calculations, the value of the force *F_Z_* was reduced by the value of the determined frictional resistance between the piston and the chamber wall. The value was determined for each pressure in the hydraulic system recorded during the tests, by taking separate measurements of the force exerted during the movement of the piston at a rate of 5 mm/s in an empty chamber.

## 3. Results

**Figure 4:**
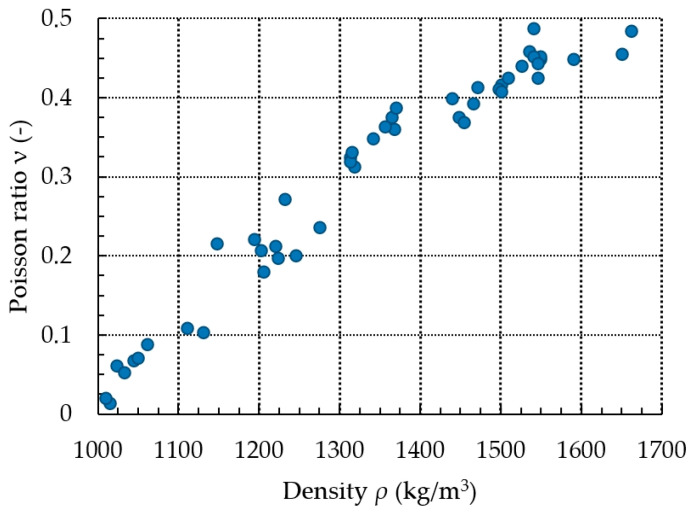
Change in Poisson ratio *ν* as a function of density *ρ*.

The test data were the basis for determining the value of the Poisson ratio *ν* for each individual test. When specimens with a density of *ρ* above 1400 kg/m^3^ were tested, the standard deviation of the density *ρ* increased compared to the tests of specimens with a density *ρ* below 1400 kg/m^3^. This was associated with the speed (5 mm/s) and the machine’s response time, since the density of 1400 kg/m^3^ with increasingly lower displacement values resulted in a considerable increase in compaction. This, in turn, translated into a greater dispersion of the obtained values of the final density of the pressure material and the values of the pressure on the side walls, at which point the system was stopped. Consequently, in the results analysis, the authors decided not to average the test results within a given series of repetitions at a fixed pressure value in the hydraulic system. The results were presented as a set of points, where a specific value of density corresponded to a given pressure and, thus, to a given value of Poisson ratio. The results obtained that illustrate the change in *ν* as a function of *ρ* are represented in the graph below (Figure 4).

**Figure 5:**
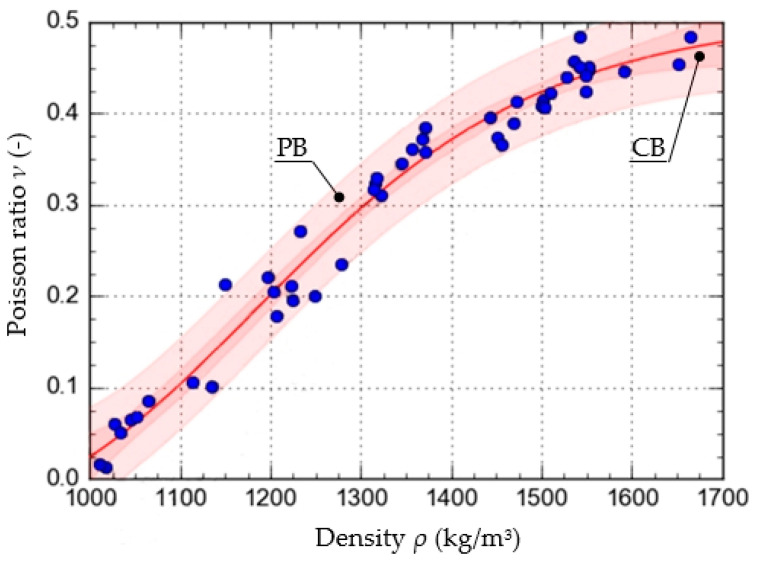
Characteristics of the change in the value of *ν* as a function *ρ*, formulated with the use of the Dose-Response Hill model.

**Figure 6:**
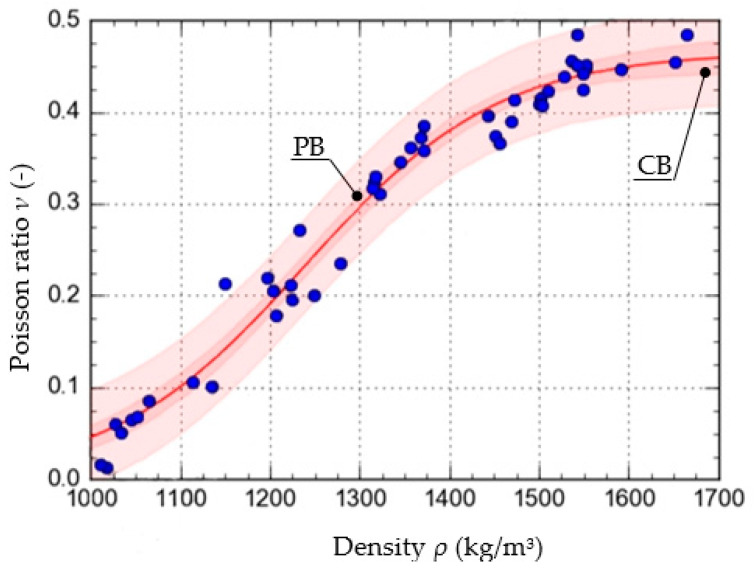
Characteristics of the change in the value of *ν* as a function *ρ*, formulated with the use of the Ratkowsky model.

**Figure 7:**
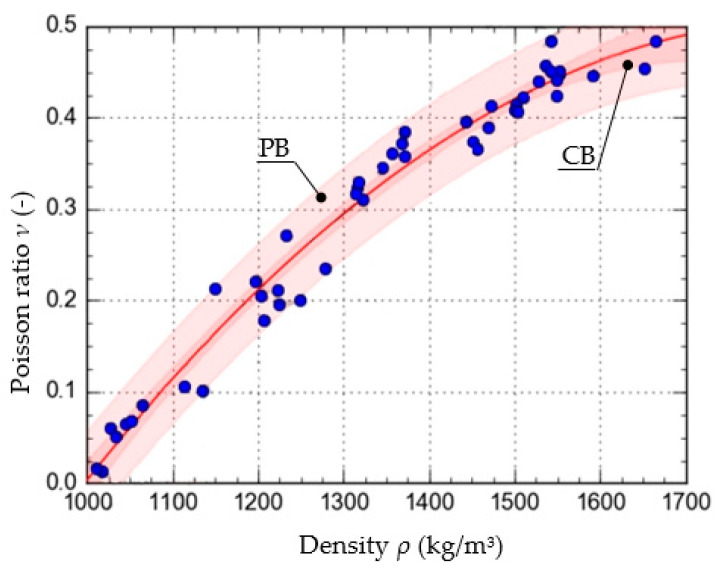
Characteristics of the change in the value of *ν* as a function *ρ*, formulated with the use of the parabolic model.

**Figure 8:**
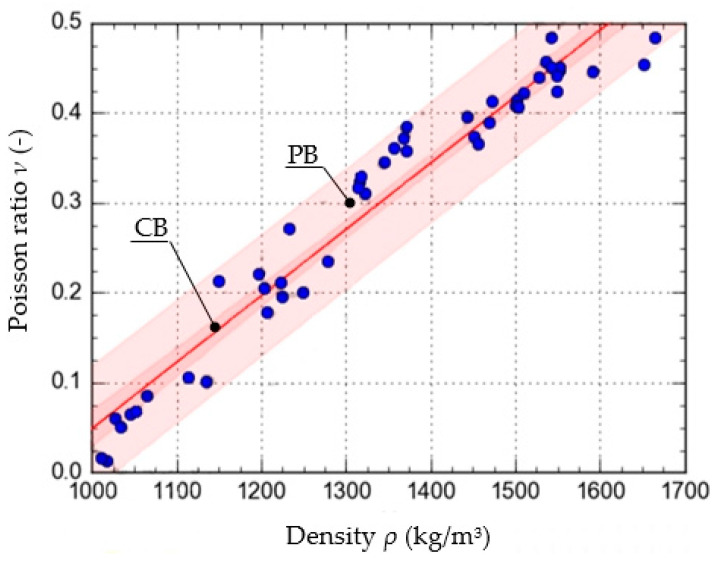
Characteristics of the change in the value of *ν* as a function *ρ*, formulated with the use of the linear model.

To determine the function that described the approximate course of the characteristic curve of the change in the value of *ν* as a function of *ρ*, calculations were carried out using the Dose-Response Hill (6), Ratkowsky (7), parabolic (8), and linear (9) models. The models, along with the values of the respective parameters, are presented as the equations given below. The curves determined from the equations are shown in Figure 5, Figure 6, Figure 7 and Figure 8, with the test results plotted as points on the graph.

(6) $$\nu =\alpha +\frac{\theta \rho^{\eta }}{\kappa^{\eta }+\rho^{\eta }}=-7.1226\cdot 10^{-2}+\frac{0.58544\;\rho^{8.223076}}{1220.08^{8.223076}+\rho^{8.22308}}\;,$$

(7) $$\nu =\frac{a}{1+e^{b-c\rho }}=\frac{0.465256}{1+e^{11.4522-9.243\cdot 10^{-3}\rho }}\;,$$

(8) $$\nu =a\rho^{2}+b\rho +c=-6.8973\cdot 10^{-7}\rho^{2}+2.559\cdot 10^{-3}\rho -1.865623\;,$$

(9) $$\nu =a\rho +b=7.389\cdot 10^{-4}\rho -0.690256\;,$$

In addition to the curves obtained from the models, the graphs also show the confidence band (CB) and the prediction band (PB).

To assess and compare the pattern of points for each of the models used, the value of the correlation coefficient and the standard error were determined. They are shown in Table 1.

The results show that the equation formulated based on the DR-Hill model has the highest correlation coefficient, 0.98754 and the lowest standard error, 0.0235. However, attention needs to be paid to slight differences between the respective models. The linear model, due to the value of the standard error, of 0.9743, was left out of consideration.

According to [32], the maximum density of dry ice is 1625 kg/m^3^. However, during preliminary tests, the team observed that the structure of a tested material with the density below 1000 kg/m^3^ was not cohesive and crumbled after being removed from the vessel. For this reason, the test results in the form of models can be used for *ρ* within the range from 1000 to 1625 kg/m^3^.

The results of the calculation of the maximum value of *ν* at the highest value of *ρ* differed slightly from each other. The results are given in Table 2.

The highest value of the Poisson ratio ν was obtained with the parabolic model and the lowest with the Ratkowsky model. The difference in the value of ν between the models was approximately 4%.

## 4. Conclusions and Results Discussion

The proposed methodology was successful at determining the value of the Poisson ratio ν for different material density values. As in the case of the previously published test results for other communited materials, the value of *ν* increased along with the increase in the degree of compaction until the limit value was reached.

Section 3 presents four mathematical models used to describe the course of the characteristic of the change in the value of ν as a function of the density of the compressed material. Three of these models are characterized by a very high value of correlation coefficient and a low value of standard error. For these models, the values of ν were determined for the maximum density of *ρ* = 1625 kg/m^3^. No significant differences were observed between them in this respect. However, it is suggested to use the derived equation that employs the Dose-Response Hill model as the first choice.

The equation obtained, in conjunction with the published results of research on the change in the value of Young’s modulus as a function of density [33] *z,* can be used in numerical studies concerning the description of compaction processes using, for example:

- The Drucker–Prager/Cap (DPC) model, to simulate the compaction of shredded natural materials [29], ceramic powders [34], pharmaceutical powders [35], and cosmetic powders [36];
- The Cam-Clay (CC) model, to simulate the compaction process for shredded-wood waste [28];
- The Mohr–Coulomb (MC) model, to simulate the densification of biomass [37] and extrusion (with the use of the elastic–plastic model [38]).

In the available research, information about the classification of compacted and extruded dry ice as a brittle material were found [39]. Furthermore, examples of the models suggesting that the indicated models were used when simulating the densification process of brittle materials were found [40], which provided examination results that were only applicable within a specific range of material density values. This follows from the maximum density value for this material, equal to 1625 kg/m^3^ [39], and the lack of cohesion of materials with a density below 1000 kg/m^3^.

Graves et al. indicated that the value of Poisson’s ratio ν is influenced by the density of the material and its temperature [41]. On the other hand, Kaufmann et al. presented the results of measuring the hardness of dry ice (carbon-dioxide ice) [42]. This team indicated in their paper that learning about the parameters of dry ice is important from the point of view of research on the planets of the solar system. The results from both of the presented studies are complementary and constitute the basis for further scientific work related to the description of the mechanical parameters of solid carbon dioxide.

The results of this study could be applied to further research involving the following:

- Numerical simulation of the compaction processes using the DPC, CC, and MC material models, for the purpose of estimating the working load;
- Numerical simulation of the extrusion processes using the elastic–plastic material model, for the purpose of estimating the working load;
- Optimization of the geometric characteristics of the tools used in the compaction and extrusion of dry ice, to increase the process efficiency;
- Analysis of the energy consumption of the dry ice palletization process with the use of a gravity roller press.

## Figures and Tables

**Table 1:** Correlation value.

Model		Correlation	Standard Error
DR-Hill	$\alpha +\frac{\theta x^{\eta }}{\kappa^{\eta }+x^{\eta }}$	0.98754	0.0235
Ratkowsky	$\frac{a}{1+e^{b-cx}}$	0.98615	0.0245
Parabolic	$ax^{2}+bc+c$	0.98657	0.0241
Linear	ax+b	0.974252	0.9743

**Table 2:** Correlation values.

Model		ν
Hill	$\alpha +\frac{\theta \rho^{\eta }}{\kappa^{\eta }+x^{\eta }}$	0.4625
Ratkowsky	$\frac{a}{1+e^{b-c\rho }}$	0.4525
Parabolic	$a\rho^{2}+b\rho +c$	0.4714

## Data Availability

Not applicable.
